# Vitamin D and Blood Parameters

**DOI:** 10.3390/biom11071017

**Published:** 2021-07-12

**Authors:** Thomas Müller, Lutz Lohse, Andreas Blodau, Katja Frommholz

**Affiliations:** 1 Department of Neurology, St. Joseph Hospital Berlin-Weissensee, Gartenstr. 1, 13088 Berlin, Germany; 2 MFSZ GmbH, Sternstr 28, 01139 Dresden, Germany; dr.lohse@yahoo.de; 3 Private Practice, Kornhausstr 85, 06846 Dessau-Roßlau, Germany; andreas.blodau@gmx.de; 4 Department of Radiology, Klinikum Frankfurt (Oder) GmbH, Müllroser Chaussee 7, 15236 Frankfurt, Germany; katja.frommholz@googlemail.com

**Keywords:** vitamin D, blood, haematocrit

## Abstract

Background: Vitamin D has a steroid- and an anabolic-resembling chemical structure. Vitamin D is essential for many processes in the human body after hydroxylation. Aims of the Study: To investigate the impact of 25-hydroxy-vitamin D plasma concentrations on the blood parameters number of erythrocytes, hematocrit, mean corpuscular hemoglobin and mean corpuscular volume. Methods: Serial assessments were done in 290 patients with multiple sclerosis and repeated after a mean interval of 245 days. A recommendation for vitamin D supplementation was given in case of a concentration lower than 20 ng/mL combined with a prescription of a formulation containing vitamin D but not vitamin K. Results: There was a fall of vitamin D in 119 subjects and a rise in 164, while no change appeared in 7 participants. When vitamin D values went down between both assessments moments, the computed increase of mean corpuscular haemoglobin was significantly lower compared with the rise of mean corpuscular haemoglobin associated with a vitamin D elevation. When vitamin D declined, the computed fall of mean corpuscular volume fall was significantly lower compared with the decrease of mean corpuscular volume, when vitamin D rose. Positive correlations were found between differences of vitamin D and mean corpuscular haemoglobin, respectively mean corpuscular volume. Inverse relations appeared between disparities of vitamin D and erythrocytes, respectively haematocrit. Conclusions: The elevation of vitamin D plasma levels provides enhanced preconditions for a better tissue oxygenation on a cellular level.

## 1. Introduction

Vitamin D is a secosteroid, which is soluble in fat. One hydroxyl group chemically characterizes vitamin D_3_ or cholecafciferol and vitamin D_2_ or ergocalciferol [1]. Cholecalciferol in the skin is the important metabolic precursor for active vitamin D generation. Sun exposure with UV radiation is the responsible component for the conversion of cholesterol to cholecalciferol [1]. Vitamin D is rare in food. Vitamin D resulting from nutrition is biologically inactive. This characteristic is similar to vitamin D derived from skin. Enzymes hydroxylate vitamin D in the liver and in the kidney. The presence of three hydroxyl groups characterizes their chemical structure. These metabolic reactions activate vitamin D and generate 1,25-dihydroxycholecalciferol or 1,25-dihydroxyvitamin D_3_ (1,25-(OH)_2_D_3_). Both may execute abundant functions in the human body [1]. This active vitamin D supports the gastrointestinal uptake of magnesium, phosphate, zinc and particularly calcium, which is essential for osteoporosis prevention and treatment. Active vitamin D strengthens the immune system and improves coronary microvascular perfusion [1,2]. Low vitamin D concentrations have been shown to be a risk factor for an elevation of the incidence and for relapses in patients with multiple sclerosis [1,3]. The nature of a causal relationship between vitamin D and MS is not explored in detail, but the beneficial effect of vitamin D supplementation on MS course is well known in the maintenance of MS patients [1]. Vitamin D supplementation is regarded as important for the immune system [1]. The measurement of 25-hydroxyvitamin D (calcifediol; 25-(OH)-vitamin D) in blood is common. It is sometimes even demanded by payers, i.e., in Germany, as precondition for the reimbursement of vitamin D supplementation. The 25-(OH)-vitamin D concentration is regarded as the best method to investigate whether the human body has enough vitamin D. Chemically, the vitamin D structure is similar to an anabolic, such as testosterone or steroids [4]. It is well known, that the chemical structure of anabolic compounds influences certain blood flow parameters, such as generation of erythrocytes [4]. As an example, the increase of red blood cell mass results from testosterone application. Androgen administration increases the generation of erythropoietin. Accordingly, the red blood cell count and blood volume go up [4]. From this point of view, one may assume that the chemical structure of 25-(OH)-vitamin D influences blood parameters, which are responsible for tissue perfusion. Essential ones are the number of erythrocytes (ERY), the hematocrit (HC) and the mean corpuscular haemoglobin concentration (MCHC), which describes the ratio between the mean corpuscular haemoglobin (MCH) and the mean corpuscular volume (MCV). The objective of this observational trial was to determine ERY, HC, MCHC, MCV, MCH and vitamin D in blood twice with an interval in between.

## 2. Materials and Methods

### 2.1. Subjects

Table 1 reports the characteristics of the 290 study participants. Multiple sclerosis patients were chosen, since they frequently perform a vitamin D supplementation.

### 2.2. Design

The levels of MCHC, MCV, MCH, HC, ERY and 25-OH-vitamin D levels were determined at moment I and moment II at a follow-up visit, which was performed within an interval of 245 ± 86.63 (days; mean ± SD) after the baseline examination. Study participants with a vitamin D plasma concentration below 20 ng/mL [50 nmol/L]) at moment I received a prescription of vitamin-D-containing formulations (Vitamin D_3_ 20.000 I.E. Depot^®^ or Dekristol^®^ 20000). The advice was given to perform a vitamin D supplementation. Vitamin-D-containing compounds with vitamin-K-containing ingredients were not recommended.

### 2.3. Methods

Blood samples were collected in EDTA-containing tubes. The determination of MCHC-, MCV-, MCH, ERY and HC was done with the Sysmex XT-2000i (XE-2100) system (Sysmex Deutschland GmbH, 22848 Norderstedt, Germany). Vitamin D measurements were undertaken with LC-MS (company: Chromsystems, Instruments & Chemicals GmbH, Am Haag 12, 82166 Gräfelfing/München; Germany). It combines reversed-phase high performance liquid chromatography (HPLC [company: Shimadzu Deutschland GmBH, Keniastr 38, 47269 Duisburg, Germany^®^] and mass spectrometry [AB-Sciex API5000, AB Sciex Germany GmbH, Landwehrstr 54, 64293 Darmstadt, Germany^®^].

### 2.4. Statistics

There was a normal distribution of data according to the Shapiro–Wilk test. The formula (value of II − value of I = difference) was employed for the calculation of differences between outcomes of moments I and II. The data were subdivided into three groups according to the computed vitamin D disparities. Cohort I included 119 subjects with a fall of vitamin D outcomes at moment II compared with moment I. Cohort II consisted of 164 participants, which experienced a rise of vitamin D values at the second assessment. Seven subjects were in cohort III with no change of vitamin D concentrations (Table 1). ANCOVA with the covariate sex and age was used for comparisons of differences between Group I, II and III. A post hoc analysis was only executed with the Tukey’s honestly significant difference (HSD) test, when ANCOVA *p* was below 0.05. It was too complex for technical reasons to perform a seasonal adjustment as a time-dependent variable of the serial vitamin D estimations [4]. The Pearson Product correlation analysis between vitamin D and blood parameters was done on an exploratory basis. A *p* value < 0.01 was regarded as significant in view of the number of participants.

### 2.5. Ethics

The study protocol and the patient informed consent form was approved by the ethics committee of the Sächsische Landesärztekammer (Number: EK-BR-72/11-1). Study participants gave written informed consent following information on the study procedures. This study was conducted in accordance with the Helsinki Declaration of 1964 and its later amendments.

## 3. Results

### 3.1. Comparisons

Table 2 describes the computed differences of all determined parameters in groups I, II and III. There were only significant variations of vitamin D, MCV and MCHC between groups I, II and III. The increase of MCHC was significant lower, when vitamin D values went down, in comparison to the MCHC rise, when vitamin D was elevated. The mean MCV fall was lower, when vitamin D declined, compared with the MCV decrease, when vitamin D rose. Changes of ERY, HC and MCH did not differ between cohorts I, II and III (Table 2). There was no impact of the covariates sex and age.

### 3.2. Correlations

The more vitamin D went up, the more MCHC and MCH rose. There were positive associations between computed differences of MCH, respectively MCHC and vitamin D (Table 3). The more vitamin D increased, the more ERY and HC went down. There were significant inverse correlations between calculated changes of vitamin D and disparities of ERY and HC (Table 3). There was no relationship between differences of vitamin D and MCV (see also Table 3). No correlations were found between the parameters sex, respectively age and all determined parameters neither in the whole cohort nor in the subgroups, subdivided according to their vitamin D change (according to Table 1) (Results not shown).

## 4. Discussion

This investigation demonstrates that changes of 25-OH-vitamin D blood levels moderately influence blood parameters, which reflect blood flow and are important for tissue oxygenation. These observed alterations show that tissue microcirculation is ameliorated with the rise of vitamin D, which confirms the initial outcomes [5]. These results suggest that vitamin D elevation enhances oxygen transport and perfusion in tissue. Vitamin D increase improves the mitochondrial function and the defence against oxidative stress, both of which play an essential pathophysiologic role in chronic neurodegeneration and neuroinflammation [6,7]. As aforementioned, there is a structural relationship between vitamin D and steroids, respectively anabolic compounds. This specific chemical structure is well known to improve oxygen perfusion [8]. Our current results confirm prior outcomes, which report associations between vitamin D deficiency and anaemia. Experimental researchers showed an enhanced survival of erythrocytes after additional vitamin D supply in mice [8]. Other investigations observed beneficial effects of body function following nutritional vitamin D supplementation in disease entities, such as atherosclerosis, diabetes mellitus or disease-related states of cognitive impairment [9,10,11,12,13]. Thus, our results provide further evidence for better preconditions for an improved tissue oxygenation on a cellular level, when higher 25-OH-vitamin D concentrations are present in blood. There was also an inverse correlation between HC and vitamin D. This association may hypothetically result from a long-term physiologic regulatory adaptation process as a consequence of the ERY changes. These alterations provide better conditions for perfusion and thus enhance the capacity for oxygen transport [8,14]. Better oxygen supply decreases the vulnerability to disease mechanisms and improves the capacity for the compensation of disease-related deficits, i.e., delirium or other cognitive disturbances [14,15]. In the past, many studies demonstrated the beneficial effects of vitamin D supplementation in various disease entities. We suggest that the efficacy of endogenous available repair mechanisms in the central nervous system may also improve, such as remyelination via the inhibition of the repulsive guidance molecule A (RGMa) pathway [16,17]. RGMa concentrations in the cerebrospinal fluid declined in progressive MS patients after continuous exposure to the retarded release steroid triamcinolone acetate. As aforementioned, the structure of this steroid resembles that of vitamin D. Disabilities, e.g., the maximum walking distance of progressive MS patients, consecutively went up, and other functions ameliorated slowly following a repeated administration. Further experimental research is urgently necessary to demonstrate that vitamin D supplementation with its steroid like structure also promotes repair via the RGMa pathway, i.e., in contrast to levodopa in patients with Parkinson’s disease [18].

This trial has several limitations. We only assessed patients twice and not exactly always after the same interval. The number of patients with the same vitamin D level (N = 7, group III) was too low to allow for any reliable conclusions for this cohort. We do not know how many patients and how long vitamin supplementation was performed or whether patients only enriched their nutrition by adding vitamin-D-containing foods. We emphasize that this described pharmacological effect of vitamin D on blood parameters may also be observed in healthy controls. MS patients were chosen as they often perform vitamin D substitution.

## 5. Conclusions

Our results underline that the change in vitamin D blood concentrations is related with an adaptation of blood parameters, which may considerably influence tissue perfusion. The rise of vitamin D provides better preconditions for an enhanced tissue oxygenation.

## Figures and Tables

**Table 1:** Characteristics of MS patients.

Age	48.28 ± 12.25
patients with a vitamin D fall	47.86 ± 13.05
patients with identical vitamin D	45.38 ± 13.03
patients with a vitamin D rise	48.71 ± 11.62
expanded disability status score	3.35 ± 2.08
Sex	77 men, 213 women
patients with a vitamin D fall	30 men, 89 women
patients with identical vitamin D	3 men, 4 women
patients with a vitamin D rise	43 men, 121 men
relapse remitting MS	195
secondary progressive MS	95 with relapses
duration of MS	3.34 ± 2.85
MS treatment	interferons: 135 (number of patients); glatiramer acetate: 37 natalizumab: 57; ocrelizumab: 9; fingolimod: 12; immune globulins: 3; teriflunamide: 22 triamcinolone (intrathecal retarded release steroid): 15

All parameters are given as mean ± standard deviation; age is given in years, MS, multiple sclerosis; duration of MS is given in years.

**Table 2:** Comparison between groups I, II, III.

I					II					III								
N = 119	mean	SD	Min	max	N = 164	mean	SD	min	max	N = 7	mean	SD	min	max	ANOVA F	I vs II	I vs III	II vs III
ERY	0.04	0.34	−0.80	1.20	ERY	−0.04	0.33	−1.08	1.20	ERY	−0.11	0.24	−0.40	0.30	1.88			
HC	0.00	0.03	−0.07	0.16	HC	−0.01	0.03	−0.08	0,14	HC	−0.01	0.02	−0.03	0.03	2.43			
MCH	0.00	0.09	−0.30	0.50	MCH	0.03	0.10	−0.30	0.40	MCH	0.03	0.08	0.03	0.08	0.19			
MCV	−0.35	3.95	−11.00	20.00	MCV	−0.63	4.02	−13.00	17.00	MCV	−0.13	1.21	−13.00	17.00	3.35	*p* < 0.05	*p* > 0.05	*p* > 0.05
MCHC	0.16	0.70	−1.70	1.60	MCHC	0.47	0.62	−0.90	2.20	MCHC	0.19	0.42	−0.70	0.50	7.77	*p* < 0.001	*p* > 0.05	*p* > 0.05
Vitamin D	−17.05	19.19	−122.70	−0.10	Vitamin D	18.46	19.84	0.10	118.50	Vitamin D	0.00	0.00	0.00	0.00	116.10	*p* < 0.001	*p* > 0.05	*p* < 0.05

ERY, number of erythrocytes; HC, hematocrit; max, maximum, min, minimum; SD, standard deviation; MCHC, ratio between mean corpuscular haemoglobin and the mean corpuscular volume; MCH, mean corpuscular haemoglobin; MCH, mean corpuscular haemoglobin, MCV, mean corpuscular volume; I, 119 patients with a fall of vitamin D; II 164 patients with a rise of vitamin D; III, 7 patients with no change of vitamin D.

**Table 3:** Correlation analysis between 25-(OH)-vitamin D blood concentrations and blood parameters.

Variable 1	Variable 2	R	P
Change of Vitamin D	Change of ERY	−0.18	0.002
Change of Vitamin D	Change of HC	−0.22	0.0002
Change of Vitamin D	Change of MCH	0.17	0.0032
Change of Vitamin D	Change of MCV	−0.08	Ns
Change of Vitamin D	Change of MCHC	0.30	*p* < 0.0001

**R:** correlation coefficient; **P**, *p*-value; change of = computed difference between both assessments according to the formula value at II − values at I; ERY, number of erythrocytes; HC, hematocrit; MCHC, ratio between mean corpuscular haemoglobin and the mean corpuscular volume; MCH, mean corpuscular haemoglobin; MCV, mean corpuscular volume; Ns, not significant.

## Data Availability

The data sets generated during and/or analysed during the current study are available from the corresponding author on reasonable request.
